# Vitamin E for Prevention of Biofilm-caused Healthcare-associated Infections

**DOI:** 10.1515/med-2020-0004

**Published:** 2019-12-26

**Authors:** Franca Vergalito, Laura Pietrangelo, Giulio Petronio Petronio, Federica Colitto, Marco Alfio Cutuli, Irene Magnifico, Noemi Venditti, Germano Guerra, Roberto Di Marco

**Affiliations:** 1 Department of Medicine and Health Sciences “V. Tiberio”, University of Molise, via De Sanctis snc, 86100 Campobasso, Italy; 2 Department of Pediatrics and Child Health, Nihon University School of Medicine, Tokyo 173-8610, Japan

**Keywords:** Medical devices, Catheter, Biofilm, Vitamin E, Alpha-Tocopheryl acetate

## Abstract

The healthcare-associated infections (HCAIs) occur in patients both in nosocomial environments and in community. More often HCAIs are associated to the use of medical devices and bacterial biofilm development on these equipments. Due to the clinical and economic relevance of this topic, new strategies for the treatment of infections caused by biofilm proliferation are unceasingly searched by scientists.

The present study investigated the role of vitamin E to reduce the biofilm formation for a larger panel of human pathogens, including strains of *Staphylococcus aureus*, *Staphylococcus epidermidis*, *Escherichia coli*, *Klebsiella pneumoniae*, *Proteus mirabilis*, *Acinetobacter baumannii*, *Pseudomonas aeruginosa* and *Pseudomonas putida*.

This potential activity was tested by placing a preparation of vitamin E (α-Tocopheryl acetate) as interface between the bacterial culture and the polystyrene walls of a 96 well plate at different concentrations of glucose, used as a biofilm enhancer.

The *Staphylococcus* genus was further investigated by spreading the vitamin E on a silicone catheter lumen and evaluating its influence on the bacterial colonization.

From our results, vitamin E has been able to interfere with bacterial biofilm and prevent *in vitro* biofilm formation. Furthermore, the ability of *Staphylococcus aureus* and *Staphylococcus epidermidis* to colonize the catheter surface decreased as a result of vitamin E application.

## 1 Introduction

Healthcare-associated infections (HCAIs) occur in patients subjected to the care process in any setting such as hospitals or patient’s own homes [1, 2, 3]. A consistent part of HCAIs are associated with the use of medical devices, and this is an important cause for patients morbidity and mortality increasing [4, 5, 6, 7, 8]. Consequently, there is a global needs to reduce the social and economic implications of HCAIs [9].

A wide range of Gram positive bacteria (e.g. *Staphylococcus aureus*, *Staphylococcus epidermidis* and *Enterococcus faecalis*), Gram negative bacteria *(*e.g. *Escherichia coli*, *Klebsiella pneumoniae*, *Proteus mirabilis, Pseudomonas aeruginosa*, *Pseudomonas putida* and *Chlamydophila pneumoniae)* and also yeasts (particularly *Candida* species) are implicated in HCAIs onset [2,3,9, 10, 11]. These microorganisms are responsible for numerous diseases like ventilator-associated pneumonia (VAP), lower respiratory tract infections (22.8 % of cases), catheter-associated urinary tract infections (CAUTIs, 17.2 % of cases) and surgical-site infections (SSIs; 15.7 % of cases) [5,12,13]. The ability of these microorganisms to grow forming biofilm makes the medical treatment of infections more difficult and in some cases leads to its failure [14,15].

A biofilm is a cellular assembly of one or more microorganisms surrounded by a complex self-produced polymeric matrix which commonly includes components from the host, such as fibrin, platelets or immunoglobulins [16, 17, 18, 19]. This complex encapsulating structure protects the microbial cells against the host immune-response system and provides a site for the adhesion of other bacterial cells [20, 21, 22]. Moreover, antibiotics may not penetrate into the biofilm layers, and also the presence of characteristic water channels inside the biofilm matrix can determine a partial antibiotics leaking together with an alteration of the environment inside the biofilm matrix that can antagonize the antibiotic action [23, 24, 25]. Finally, mechanisms of plasmid gene transferring may conduce to the onset of resistant bacterial strains which can generate molecules that antagonize the antibiotics inducing a reduction of their therapeutic role [26,27]. Indeed, there are many scientific evidences that bacteria living in a mature biofilm can tolerate antibiotics concentrations 10-1000 higher compared to planktonic bacteria [24,28,29].

Due to the clinical and economical relevance of this topic, new strategies for the treatment of infections caused by the biofilm proliferation are unceasingly searched by scientists [30,31].

The evidence of a potential role of vitamins as antibiofilm/antimicrobial agent is not recent. In 1999 Habash et al. investigated the role of vitamin C in the reduction of adhesion of some uropathogens onto biomaterials utilized within the urinary tract [32].

More recent studies hypothesized a similar function for vitamin E. The capability of *Staphylococcus* ssp. and *E. coli* to form biofilm onto different biomaterials blended with vitamin E was investigated. Although not consistent results have been obtained by different studies due to the different methods used and different pathogens tested [1,33, 34, 35, 36].

Therefore, the present study investigated the role of vitamin E to reduce the biofilm formation for a larger panel of human pathogens, including *S. aureus*, *S. epidermidis*, *E. coli*, *K. pneumoniae*, *P. mirabilis*, *A. baumannii*, *P. aeruginosa* and *P. putida*. This activity was tested placing a preparation of vitamin E as an interface between the bacterial culture and the polystyrene wells of a 96 well plate at different concentrations of glucose, used as a biofilm formation enhancer. Moreover, bacterial species which commonly cause the infections associated with the use of urinary catheters belonging to the *Staphylococcus* genus were further investigated. More precisely, the vitamin E was directly spread on a silicone catheter lumen to evaluate its influence on the bacterial colonization and biofilm formation.

## 2 Methods

Ethical approval: the conducted research is not related to either human or animals use

### 2.1 Chemicals

Tryptic Soy Broth (Sigma Aldrich), glucose (Sigma Aldrich), vitamin E (α-Tocopheryl acetate) (≥96%, 0.95 g/ml, Sigma Aldrich), 96 well cell culture plate flat bottom (Orange Scientific), NaCl (Sigma Aldrich), methanol solution (for HPLC, ≥99.9%, Sigma-Aldrich), glacial acetic acid (≥99.85%, Sigma Aldrich), 2% crystal violet solution (from the Gram color kit Liofilchem).

### 2.2 Tested strains

*Staphylococcus aureus* ATCC 29213, *Staphylococcus epidermidis* ATCC 12228, *Escherichia coli* ATCC 11775, *Klebsiella pneumoniae* ATCC 700603, *Proteus mirabilis* ATCC 29906, *Acinetobacter baumannii* ATCC 19606, *Pseudomonas aeruginosa* ATCC 27853 and *Pseudomonas putida* ATCC 12633.

### 2.3 *In vitro* toxicity test

The *in vitro* toxicity of vitamin E was estimated through a microdilution assay as suggested by Tintino et al. with some modifications [37]. Tested bacterial strains were grown in Tryptic Soy Broth (TSB) and TSB with 1 and 2.5% glucose, at 37°C for 24 hours. All cultures were diluted to the cell concentration of 10^8^ CFU/ml, and 100 ul serial dilutions were prepared in triplicates in the 96 well microtiter plates. Vitamin E was added to the subsequent wells at the range of final concentration from 100 to 400 mg/ml, with concentration increments of 100 mg/ml. Also, triplicates of different media and *inocula* only were prepared in plate wells as controls. The plates were incubated at 37°C for 24 hours and bacterial growth was determined plating proper dilutions of the bacterial cultures on 1.8% agarose TSB by colony counting after 24 hours of incubation. The highest concentration of vitamin E that did not interfere with the growth of all the tested bacterial strains, i.e. the concentration of 200 mg/ml, was used in subsequent *in vitro* experiments for biofilm eradication.

### 2.4 *In vitro* biofilm formation assay

Cultures of tested strains were grown in TSB at 37°C for 24 hours, centrifuged at 3000 rpm for 10 min and the pellet was re-suspended to the final concentration of 10^8^ CFU/ml in 2 ml of fresh TSB, TSB with 1 and 2.5% glucose, respectively. 200 ul aliquots of each culture were added to a polystirene 96 well plate as well as 200 ul triplicates of TSB and TSB with 1, and 2.5 % glucose without inocula were prepared as controls. Vitamin E was added to all wells at the final concentration of 200 mg/ml. Another plate was generated with the same samples and controls without vitamin E. Both plates were incubated at 37°C for 24 hours. After incubation time the biofilm production was estimated through the method proposed by Stepanović et al. with some modification [38]. The wells were emptied and washed three times using 250 ul of 0.9% NaCl. Therefore, 200 ul of methanol solution were added incubating for 15 min to fix the cells which eventually adhered to the tubes. The methanol solution was discarded, and the plates were dried under the biological laminar flow in upset-down position. Subsequently, 200 ul of crystal violet solution were added to wells and maintained in incubation for 5 min. Then, the excess of staining was removed washing the plates under a moderate tap water flow.

The dye bound to the adherent cells was resolubilized with 160 μl of 33% glacial acetic acid and the Optical Density (O.D.) at 570 nm for each well was measured using the VICTOR X5 multilabel plate reader (Perkin-Elmer) [38, 39, 40]. For each considered strain the comparison between O.D. values obtained with/without vitamin E was performed, and consequently, the strains attitude to form biofilm under the presence/absence of vitamin E was established.

Furthermore, the effect of two different glucose concentrations was assessed adding 1% or 2.5% sugar to TSB during bacterial growth.

For each strain, the O.D. values obtained in all tested conditions under vitamin E presence were compared to those obtained under vitamin E absence and considered as the maximum rate of their biofilm formation capability (100%). Therefore, the percentage reduction of biofilm formation under vitamin E influence was calculated as the mean value between the percentage reduction in all tested conditions.

The biofilm producing strains more susceptible to vitamin E action were subjected to further investigation to evaluate the possible application of vitamin E for the prevention of biofilm formation on the surface of medical devices.

### 2.5 *In vitro* biofilm formation on the surface of medical devices

The influence of vitamin E on the ability of the *Staphylococcus* strains to form biofilm layers on the lumen of silicone urinary catheters was also investigated.

The lumen of silicone catheters sections (continuous irrigation balloon catheters DBK-Dufour UROMED) of 2 cm in length were previously homogeneously layered by the same concentration of vitamin E used in the *in vitro* biofilm assay (200 mg/ml) and subsequently covered by 200 ul of strains suspensions (10^8^ cells/ml) and media for which in this study the more relevant effect of the vitamin E was observed. Also, controls were prepared applying 200 ul of each medium only on the surface of catheter sections with vitamin E layer, as well as sections of the device with vitamin E only were prepared. All catheter sections were incubated at 37°C for 24 hours.

After incubation time sections were washed and stained as described above and the stain was re-suspended using 200 ul of 33% glacial acetic acid. The stain suspensions were distributed to a 96 well plate for the O. D. measurement as described above.

The effect of the vitamin E layer in reducing the biofilm formation of *Staphylococcus* strains on the catheter surface was evaluated: the O.D. values obtained for the sections inoculated with bacteria and pre-treated with the vitamin E were compared to those obtained for sections not previously layered by vitamin E.

## 3 Statistical analysis

Statistical data analysis was performed using SigmaPlot version 12.0, from Systat Software, Inc. (San Jose California, USA). For each strain firstly the normality of data was evaluated through Shapiro-Wilk test. The values groups resulted as normal distributed were subjected to the ANOVA analysis with post-hoc Tukey HSD test (p<0.05). The values series not normally distributed were statistically analysed using the not parametric Kruskal-Wallis test FDR corrected (p<0.05).

## 4 Results

### 4.1 *In vitro* toxicity test

Vitamin E concentration of 200 mg/ml did not interfere with the growth of all tested bacteria (data not shown) and therefore this dosage was used for the *in vitro* biofilm experiments.

### 4.2 *In vitro* biofilm formation

**Figure 1:**
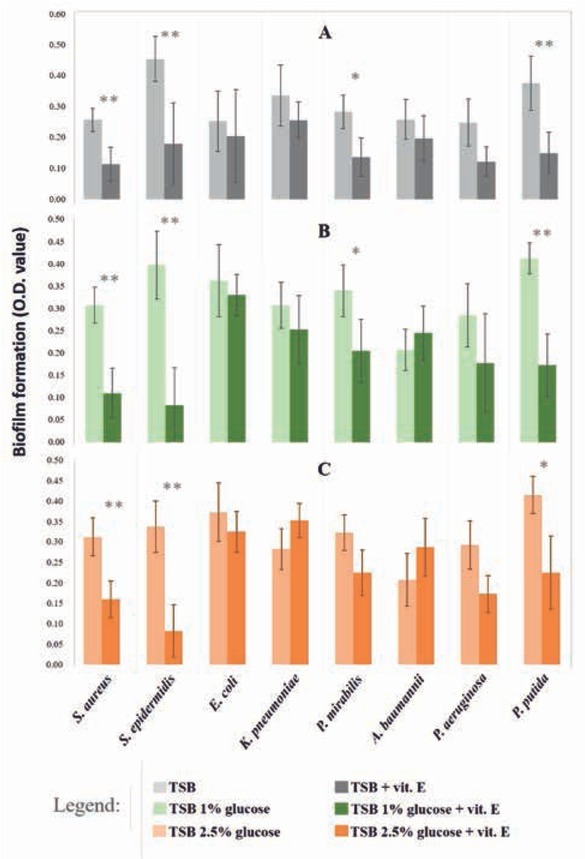
Influence of vitamin E and glucose on the biofilm formation by common human pathogens. Bars indicate the biofilm formed by strains in A) TSB with (dark grey) and without (light grey) vitamin E B) TSB 1% glucose with (dark green) and without (light green) vitamin E C) TSB 2.5% glucose with (dark orange) and without (light orange) vitamin E. The estimation of biofilm formed is based on the optical density of solutions obtained through the resuspension of the stain (see section Methods). Asterisks indicate significance of these comparisons, with «*» for p<0.05 and «**» for p<0.01 (ANOVA and Kruskal-Wallis test, with p<0.05).

The inhibitory action of vitamin E (α-Tocopheryl acetate) on biofilm formation was evaluated for all tested strains. Although for *E. coli*, *K. pneumoniae*, *A. baumannii* and *P. aeruginosa* no significant results were obtained (ANOVA analysis with post-hoc Tukey HSD test and not parametric Kruskal-Wallis test FDR corrected, p<0.05), a reduction of biofilm formation was observed at least in one of tested conditions (Figure 1). In detail, both with and without glucose adding a reduction trend between 9-19% was calculated for *E. coli* as well as 38-51% for *P. aeruginosa* (Figure 1, Table 1). An inhibitory effect of vitamin E alone and in association with 1% glucose was detected for *K. pneumoniae* with reduction percentages of 24 and 18% respectively (Figure 1, Table 1). Finally, for *A. baumannii* the reduction trend was observed only by application of vitamin E alone with a biofilm decreasing of 24% (Figure 1, Table 1).

On the contrary, for *S. aureus*, *S. epidermidis* and *P. putida* the application of vitamin E both with and without glucose adding caused a significant decreasing of biofilm formation meanly larger than 50%. More precisely, in presence of vitamin E alone and in association with 1 and 2.5% of glucose the biofilm of *S. aureus* was reduced of 56, 64 and 49% respectively, for *S. epidermidis* of 61, 79 and 76% and for *P. putida* of 61, 58 and 46% (Figure 1, Table 1).

Moreover, for *P. mirabilis* the biofilm formation resulted significantly reduced of 52 and 40% applying vitamin E alone and in association with the lowest glucose concentration; a trend of biofilm reduction was also observed when vitamin E was applied with the highest glucose concentration even though this variation resulted not significant (Figure 1, Table 1).

Regarding the effect of glucose in association with vitamin E on biofilm formation, we verified that glucose was not significantly influential in biofilm reduction when associated with vitamin E. No significant differences were obtained between vitamin E treatments with and without glucose adding, whereas significant variations were obtained with vitamin E adding respect to the media without it (ANOVA analysis with post-hoc Tukey HSD test and not parametric Kruskal-Wallis test FDR corrected, p<0.05).

**Table 1:** Variation of biofilm formation in media added with vitamin E respect to media not added by vitamin E. For each strains is reported the percentage of reduction (red bars) or increasing (blue bars) of biofilm formation in media added with vitamin E respect to the same media not added with vitamin E. The negative and positive numbers indicate percentages of decrement or increment of biofilm respectively.

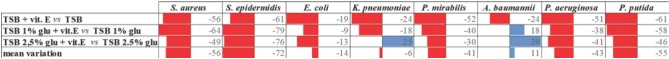

### 4.3 *In vitro* biofilm formation on the surface of medical devices

**Figure 2:**
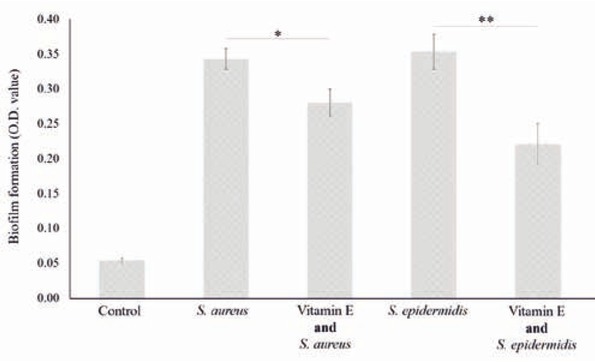
Effect of vitamin E pre-treatment of catheter surfaces on the biofilm formation ability of *Staphylococcus* strains. «Control» stands for medium not added with vitamin E and not inoculated by considered strains. Statistical significance of interesting comparisons is indicated by asterisks, with «*» for p< 0.05 and «**» for p<0.01 (ANOVA, post-hoc Tukey HSD, p<0.05).

The influence of vitamin E on the ability of both *Staphylococcus* strains to colonize the lumen of catheter section was evaluated. The O.D. values measured for suspensions from catheter sections incubated into *Staphylococcus* cultures and pre-treated with vitamin E were lower than the ones measured for sections untreated. More precisely, the ability of *S. aureus* and *S. epidermidis* to colonize the catheter surface decreased 17% and 36% (Student t-test, p<0,05) respectively with the application of vitamin E (Figure 2).

## 5 Discussion

Biofilm forming microorganisms are commonly responsible for HCAIs. More relevant, into the biofilm assembles these bacteria are protected by the action of antibiotics and this leads to the failure of clinical treatments or at least to the prolonging of the pathologic conditions.

In this context, *S. aureus* and *S. epidermidis* are Gram positive biofilm producer *bacteria* that play an important role in HCAIs onset [41,42]. Although the infection caused by these pathogens included a low percentage of HCAIs, it should not be underestimated. Indeed, in urinary tract infections (UTI) a significant relationship between biofilm formation by *S. aureus* strains and some antibiotic resistance have been demonstrated [43]. For this reason, it is necessary to intervene in the initial phases of biofilm formation to prevent bacterial colonization.

In last years, alternative approaches for the treatment of biofilm-related infections appear as necessity firstly for the treatment of the most common HCAIs due to the use of urinary catheters [44, 45, 46, 47]. Many natural substances and synthetic molecules compatible with both human physiology and the constitutive materials of medical devices, and potentially capable of interfering with pathogen attachment to surfaces have been evaluated [46,47].

In the present study we analyzed the *in vitro* effects of vitamin E, well known for its beneficial activity on human and animals, in biofilm formation evaluating its ability to interfere with bacterial colonization of medical devices by a large panel of human pathogens implicated in HCAIs onset including *S. aureus, S. epidermidis, E. coli, K. pneumoniae, P. mirabilis, A. baumannii, P. aeruginosa* and *P. putida*.

Results related to biofilm formation have shown that, although the concentration used does not inhibit bacterial growth (200 mg/ml), it is still able to reduce the biofilm production capacity of all the strains tested, with a variable efficacy between strains without any correlation to the Gram negative or positive group. The low antibacterial activity, i.e. low toxicity, was confirmed by Bidossi et al. [48] testing a soluble form of vitamin E (α-tocopheryl phosphate) against different strains of *S. aureus*, *S. epidermidis* and *P.aeruginosa* and reporting MIC values very close to one of the liposoluble forms used in our study (100-200 mg/ml) [48]. The sensible value variation may be likely due to the different strains tested and to the enhanced solubility of the phosphate form compared to α-Tocopheryl acetate [48]. Moreover, a recent study by Naguib et al. [49] demonstrated the low antibacterial activity of vitamin E alone compared to its combination with norfloxacin or ceftazidemime that increased antimicrobial sensitivity to *B. cenocepacia* and *P. aeruginosa* through the inhibition of the bacterial lipocalin antibiotic binding [49].

Furthermore, the effect of different glucose concentrations (1% and 2.5% w/v) on the action of vitamin E has provided interesting results. Although the role of glucose as biofilm enhancer is well known, we verified that no significant differences could be appreciated between vitamin E treatments with and without glucose adding for all tested strains. This suggests that the inhibition of biofilm formation was ascribable mainly to the vitamin E action able to mask the enhancing effect of glucose.

Moreover, although the increased biofilm formation by staphylococcal species has been reported in response to increased glucose concentrations [50], our results suggest a potential synergistic and inhibitory effect of vitamin E/glucose at low concentrations tested.

Regarding the ability of vitamin E to prevent *Staphylococcus* strains adhesion on surfaces, our data have confirmed the results of previous studies [48,51] even though conflicting results have been observed in these studies due to the different materials and strains tested. Results obtained with polystyrene plates and silicone catheters highlight the importance of the material constituting the devices when the formation of biofilms is tested *in vitro*. The biofilm reduction percentage of *S. aureus* and S. *epidermidis* in silicone cathers, under the same experimental conditions, is significantly lower compared to polystyrene plates.

These results obtained for these specific strains related to glucose addition could be explained by a hypothesis formulated by Campocia et al. [34]; according to the authors the surface physico-chemical properties of biomaterials variably affect the adherence of the microorganism and the formation of biofilms, while the vitamin E action determines a conditioning of the bacterial surfaces, rather than of the biomaterial surfaces.

This hypothesis seems to be confirmed by numerous studies conducted on the antibiofilm action of polymers made with the addition of vitamin E against *Staphylococcus* spp. which is demonstrated as the reduced bacterial adhesion was not dependant by the adding of vitamin E to all the polymers tested [35,36,52,53].

Concluding, our results together showed that vitamin E could significantly reduce *in vit*ro biofilm formation of some bacterial strains generally considered to be the most frequent pathogens responsible for HCAIs onset.

Furthermore, it was found that *S. aureus* and *S. epidermidis* were more susceptible to the action of vitamin E. A potential role of vitamin E for prevention of biofilm formation on the surface of medical devices was also demonstrated.

Although in our experiments an antimicrobial effect of vitamin E at high concentrations was observed, further studies are needed to better clarify the mechanisms, the spectrum of activity and the influence on that of glucose adding.

However, our findings together prospect the promising use of vitamin E as coating molecule to prevent implant-associated infections and improve post-operative course.

## Abbreviations

HCAIs: (healthcare-associated infections)
VAP: (ventilator-associated pneumonia)
CAUTIs: (catheter-associated urinary tract infections)
SSIs: (surgical-site infections)
TSB: (Tryptic Soy Broth)
O.D.: (Optical Density)
UTI: (urinary tract infections)

## References

[1] van Kleef E, Robotham J V., Jit M, Deeny SR, Edmunds WJ (2013). Modelling the transmission of healthcare associated infections: A systematic review. BMC Infect Dis.

[2] Khan HA, Baig FK, Mehboob R (2017). Nosocomial infections: Epidemiology, prevention, control and surveillance. Asian Pac J Trop Biomed.

[3] Haque M, Sartelli M, McKimm J, Bakar MA (2018). Health care-associated infections – An overview. Infect Drug Resist.

[4] Donlan R (2008). Biofilms on Central Venous Catheters: Is Eradication Possible?. Curr Top Microbiol Immunol.

[5] Percival SL, Suleman L, Vuotto C, Donelli G (2015). Healthcare-Associated infections, medical devices and biofilms: Risk, tolerance and control. J Med Microbiol.

[6] Ripabelli G, Salzo A, Mariano A, Sammarco ML, Tamburro M (2019). Healthcare-associated infections point prevalence survey and antimicrobials use in acute care hospitals (PPS 2016–2017) and long-term care facilities (HALT-3): a comprehensive report of the first experience in Molise Region, Central Italy, and targeted in. J Infect Public Health.

[7] Ripabelli G, Sammarco ML, Scutellà M, Felice V, Tamburro M Carbapenem-Resistant KPC- and TEM-Producing *Escherichia coli* ST131 Isolated from a Hospitalized Patient with Urinary Tract Infection: First Isolation in Molise Region, Central Italy, July 2018. Microb Drug Resist.

[8] Lo Bue M. A., Di Marco R. M., Milazzo I., Nicolosi D., Calì G., Rossetti GB B. (2008). Microbiological and clinical periodontal effects of fixed orthodontic appliances in pediatric patients. New Microbiol.

[9] Stone PW (2009). Economic burden of healthcare-associated infections: An American perspective. Expert Rev Pharmacoeconomics Outcomes Res.

[10] Donlan RM (2001). Biofilm Formation: A Clinically Relevant Microbiological Process. Clin Infect Dis.

[11] Revelas A (2012). Healthcare - associated infections: A public health problem. Niger Med J.

[12] Hopkins S, Karen S, Lisa S Healthcare Protection Agency (2012) English National Point Prevalence Survey on Healthcare-associated Infections and Antimicrobial Use, 2011: Preliminary data. Healthc Assoc Infect Guid data Anal. 2012;1–140.

[13] Ripabelli G, Tamburro M, Guerrizio G, Fanelli I, Flocco R, Scutellà M (2018). Tracking Multidrug-Resistant *Klebsiella pneumoniae* from an Italian Hospital: Molecular Epidemiology and Surveillance by PFGE, RAPD and PCR-Based Resistance Genes Prevalence. Curr Microbiol.

[14] Römling U, Balsalobre C (2012). Biofilm infections, their resilience to therapy and innovative treatment strategies. J Intern Med.

[15] Baldassarri L, Creti R, Recchia S, Imperi M, Facinelli B, Giovanetti E (2006). Therapeutic failures of antibiotics used to treat macrolide-susceptible *Streptococcus pyogenes* infections may be due to biofilm formation. J Clin Microbiol.

[16] Flemming H-C, Wingender J (2010). The biofilm matrix. Nat Rev Microbiol.

[17] Lindsay D, von Holy A (2006). Bacterial biofilms within the clinical setting: what healthcare professionals should know. J Hosp Infect.

[18] Percival SL, Hill KE, Malic S, Thomas DW, Williams DW (2011). Antimicrobial tolerance and the significance of persister cells in recalcitrant chronic wound biofilms. Wound Repair Regen.

[19] Signorelli SS, Anzaldi M, Libra M, Navolanic PM, Malaponte G, Mangano K (2016). Plasma Levels of Inflammatory Biomarkers in Peripheral Arterial Disease: Results of a Cohort Study. Angiology.

[20] Domenech M, Ramos-Sevillano E, García E, Moscoso M, Yuste J (2013). Biofilm formation avoids complement immunity and phagocytosis of *Streptococcus pneumoniae*. Infect Immun.

[21] Leid JG (2009). Bacterial biofilms resist key host defenses. Microbe.

[22] Blandino G, Fazio D, Di Marco R (2008). Probiotics: Overview of microbiological and immunological characteristics. Expert Rev Anti Infect Ther.

[23] Steward PS, Costdrton JW (2001). Antibiotic resistance of baqteria in biofilms. Lancet.

[24] Olsen I (2015). BiofilmBiofilm-specific antibiotic tolerance and resistance. Eur J Clin Microbiol Infect Dis.

[25] Balcázar JL, Subirats J, Borrego CM (2015). The role of biofilms as environmental reservoirs of antibiotic resistance. Front Microbiol.

[26] Hall CW, Zhang L, Mah TF (2017). PA3225 is a transcriptional repressor of antibiotic resistance mechanisms in *Pseudomonas aeruginosa*. Antimicrob Agents Chemother.

[27] Ridenhour BJ, Metzger GA, France M, Gliniewicz K, Millstein J, Forney LJ (2017). Persistence of antibiotic resistance plasmids in bacterial biofilms. Evol Appl.

[28] El-Gebaly E, Essam T, Hashem S, El-Baky RA (2012). Effect of levofloxacin and vitamin C on bacterial adherence and preformed biofilm on urethral catheter surfaces. J Microb Biochem Technol.

[29] Macià MD, Rojo-Molinero E, Oliver A (2014). Antimicrobial susceptibility testing in biofilm-growing bacteria. Clin Microbiol Infect.

[30] Shorr AF, Haque N, Taneja C, Zervos M, Lamerato L, Kothari S (2010). Clinical and economic outcomes for patients with health care-associated *Staphylococcus aureus* pneumonia. J Clin Microbiol.

[31] Wu H, Moser C, Wang HZ, Høiby N, Song ZJ (2015). Strategies for combating bacterial biofilm infections. Int J Oral Sci.

[32] Habash MB, Van der Mai HC, Busscher HJ, Reid G (1999). The effect of water, ascorbic acid, and cranberry derived supplementation on human urine and uropathogen adhesion to silicone rubber. Can J Microbiol.

[33] Banche G, Allizond V, Bracco P, Bistolfi A, Boffano M, Cimino A (2014). Interplay between surface properties of standard, vitamin E blended and oxidised ultra high molecular weight polyethylene used in total joint replacement and adhesion of *Staphylococcus aureus* and *Escherichia coli*. Bone Jt J.

[34] Campoccia D, Visai L, Renò F, Cangini I, Rizzi M, Poggi A (2015). Bacterial adhesion to poly-(D,L)lactic acid blended with vitamin E: Toward gentle anti-infective biomaterials. J Biomed Mater Res - Part A.

[35] Gómez-Barrena E, Esteban J, Molina-Manso D, Adames H, Martínez-Morlanes MJ, Terriza A (2011). Bacterial adherence on UHMWPE with vitamin E: An in vitro study. J Mater Sci Mater Med.

[36] Kyomoto M, Shobuike T, Moro T, Yamane S, Takatori Y, Tanaka S (2015). Prevention of bacterial adhesion and biofilm formation on a vitamin E-blended, cross-linked polyethylene surface with a poly(2-methacryloyloxyethyl phosphorylcholine) layer. Acta Biomater.

[37] Tintino SR, Morais-Tintino CD, Campina FF, Pereira RL, Costa M do S, Braga MFBM (2016). Original article : ACTION OF CHOLECALCIFEROL AND ALPHA-TOCOPHEROL ON. EXCLI J.

[38] Stepanović S, Vuković D, Dakić I, Savić B, Švabić-Vlahović M (2000). A modified microtiter-plate test for quantification of staphylococcal biofilm formation. J Microbiol Methods.

[39] Stepanović S, Ćirković I, Ranin L, Švabić-Vlahović M (2004). Biofilm formation by *Salmonella* spp. and *Listeria monocytogenes* on plastic surface. Lett Appl Microbiol.

[40] Pietrangelo L, Bucci A, Maiuro L, Bulgarelli D, Naclerio G (2018). Unraveling the composition of the root-associated bacterial microbiota of *Phragmites australis* and *Typha latifolia*. Front Microbiol.

[41] Soto SM (2014). Importance of Biofilms in Urinary Tract Infections: New Therapeutic Approaches. Adv Biol.

[42] Delcaru C, Alexandru I, Podgoreanu P, Grosu M, Stavropoulos E, Chifiriuc MC (2016). Microbial biofilms in urinary tract infections and prostatitis: Etiology, pathogenicity, and combating strategies. Pathogens.

[43] Yousefi M, Pourmand MR, Fallah F, Hashemi A, Mashhadi R, Nazari-Alam A (2016). Characterization of *Staphylococcus aureus* biofilm formation in urinary tract infection. Iran J Public Health.

[44] Jacobsen SM, Stickler DJ, Mobley HLT, Shirtliff ME (2008). Complicated catheter-associated urinary tract infections due to *Escherichia coli* and *Proteus mirabilis*. Clin Microbiol Rev.

[45] Nicolle LE (2014). Catheter associated urinary tract infections. Antimicrob Resist Infect Control.

[46] Siddiq DM, Darouiche RO (2012). New strategies to prevent catheter-associated urinary tract infections. Nat Rev Urol.

[47] Singha P, Locklin J, Handa H, Author AB (2017). A Review of the Recent Advances in Antimicrobial Coatings for Urinary Catheters Graphical Abstract HHS Public Access Author manuscript. Acta Biomater.

[48] Bidossi A, Bortolin M, Toscano M, De Vecchi E, Romanò CL, Mattina R (2017). In vitro comparison between α-tocopheryl acetate and α-tocopheryl phosphate against bacteria responsible of prosthetic and joint infections. PLoS One.

[49] Naguib MM, Valvano MA (2018). Vitamin E Increases Antimicrobial Sensitivity by Inhibiting Bacterial Lipocalin Antibiotic Binding. mSphere.

[50] Waldrop R, McLaren A, Calara F, McLemore R (2014). Biofilm Growth Has a Threshold Response to Glucose in Vitro. Clin Orthop Relat Res.

[51] Banche G, Bracco P, Allizond V, Bistolfi A, Boffano M, Cimino A (2015). et al. Do Crosslinking and Vitamin E Stabilization Influence Microbial Adhesions on UHMWPE-based Biomaterials? Clin Orthop Relat Res.

[52] Williams DL, Vinciguerra J, Lerdahl JM, Bloebaum RD (2015). Does Vitamin E-blended UHMWPE Prevent Biofilm Formation?. Clin Orthop Relat Res.

[53] Banche G, Bracco P, Bistolfi A, Allizond V, Boffano M, Costa L (2011). Vitamin e blended Uhmwpe may have the potential to reduce bacterial adhesive ability. J Orthop Res.

